# Supplementary material for: Bacterial Volatiles Known to Inhibit Phytophthora infestans Are Emitted on Potato Leaves by Pseudomonas Strains
Source: Microorganisms. 2022 Jul 26;10(8):1510. doi: 10.3390/microorganisms10081510 (PMC9394277; doi:10.3390/microorganisms10081510)
Supplement: Supplementary file 1 [file microorganisms-10-01510-s001.zip › Supplementary Figure S1&Table S1.pdf]

microorganisms

MDPI

Supplementary material for

Brief Report

Bacterial volatiles known to inhibit *Phytophthora infestans* are emitted on potato leaves by *Pseudomonas* strains

Aurélie Gfeller, Pascal Fuchsmann, Mout De Vrieze, Katia Gindro and Laure Weisskopf

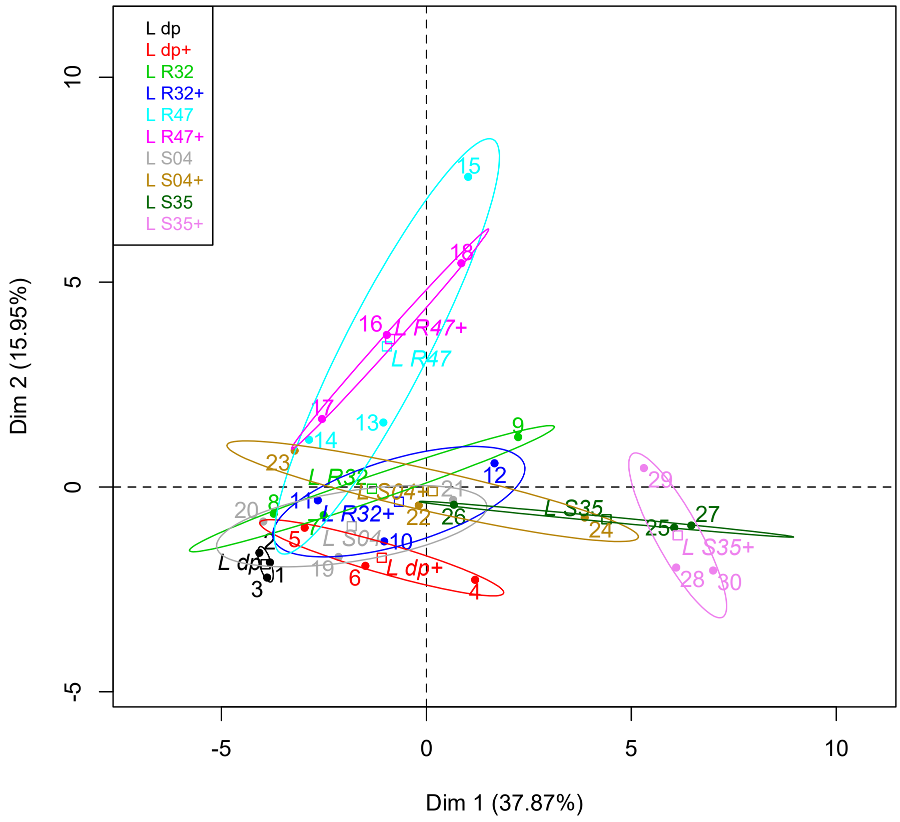

Figure S1 Principal component analysis of the volatiles emitted by leaves inoculated with *Pseudomonas* strains in presence and absence of *P. infestans*. Score Plot of PC1 and PC2 of potato leaves non-inoculated (N) or inoculated with different *Pseudomonas* strains (R32, R47, S04, S35) in presence and absence of *P. infestans* following PCA. Superimposed on the plot are mean scores on the components for qualitative variables that are included in the PCA command and the coloured ellipses illustrating the 95% confidence interval with each score. (L, leaf; dp, dipped in water (non-inoculated leaves); +, infected with *P. infestans*).

Table S1 Abundance of bacteria on leaves 2h and 120h after inoculation

Leaves were inoculated with washed cells of *Pseudomonas* R47, R32, S04, S35, or left uninoculated (CT). *P. infestans* was either inoculated (+) or not (-) and colony forming units (CFU) were counted by serially diluting ground leaves and plating them on selective media.

Strain	*P. infestans*	CFU/leaf (t= 2h)	CFU/leaf (t= 120h)
R47	-	5,00E+06	1,20E+11
R47	+	3,70E+07	5,00E+11
R32	-	6,30E+07	1,40E+11
R32	+	8,50E+07	9,00E+10
S04	-	5,80E+07	5,00E+11
S04	+	4,50E+07	1,50E+11
S35	-	8,00E+06	8,00E+11
S35	+	2,70E+07	3,00E+11
CT	-	-	0
CT	+	-	0

Table S2 List of volatile organic compounds identified in leaf samples. All compounds differentially present from the blank are presented. Compounds are putatively identified by comparison with NIST database; N= absence of treatment; Y: presence of treatment; R1, R2, R3= repetition 1,2,3 ; N=3

Please see separate Excel file
